# Sensitization of neuroblastoma for vincristine-induced apoptosis by Smac mimetic LCL161 is attended by G2 cell cycle arrest but is independent of NFκB, RIP1 and TNF-α

**DOI:** 10.18632/oncotarget.21193

**Published:** 2017-09-23

**Authors:** Doerte Langemann, Magdalena Trochimiuk, Birgit Appl, Patrick Hundsdoerfer, Konrad Reinshagen, Georg Eschenburg

**Affiliations:** ^1^ Department of Pediatric Surgery, University Medical Center Hamburg-Eppendorf, Hamburg, Germany; ^2^ Department of Pediatric Oncology/Hematology, Charité - Universitätsmedizin Berlin, Berlin, Germany

**Keywords:** neuroblastoma, LCL161, vincristine, NFκB, G2 cell cycle arrest

## Abstract

We demonstrated sensitization for chemotherapy by Smac mimetic (SM) LCL161, a potent antagonist of inhibitor of apoptosis proteins (IAP), in neuroblastoma (NB). Vinca alkaloids, particularly vincristine (VCR), displayed the strongest impact on inhibition of proliferation and apoptosis induction in combination with LCL161. The underlying signaling pathways remain elusive, though. LCL161 induces a quick degradation of cellular IAP 1 (cIAP-1). Combination of LCL161 with VCR had only marginal effects on X-linked IAP (XIAP) protein expression. Cell death is accompanied by activation of intrinsic (caspase-9 and MMP) and extrinsic (caspase-8) pathways of apoptosis, repression of migratory potential and cell cycle arrest in G2 phase.

LCL161-induced cIAP degradation leads to activation of non-canonical and blockade of canonical NF-κB pathways but not induction of apoptosis. Surprisingly NF-κB and TNF-α signaling is negligible for VCR- and VCR/LCL161-induced apoptosis since chemical inhibition of NF-κB using BAY-7085 and PBS-1086, as well as application of TNF-α blocking antibody Humira (adalimumab) has no relevant effect on cell death. Recently formation of a TNF-α-independent complex (ripoptosome) consisting of RIP1, FADD and caspase-8 following IAP inhibition by SM has been described. However, targeting of RIP1 by Necrostatin was not sufficient to influence apoptosis induced by VCR/LCL161.

## INTRODUCTION

The development of more effective therapeutic approaches for neuroblastoma, the most common solid extracranial tumor during infancy and childhood, is one of the most relevant issues in pediatric oncology [1, 2]. Significant improvements in therapy including megatherapy with blood stem cell exchange or immunotherapy were developed in recent years; nevertheless, prognosis for high-risk and late-stage disease is still poor as metastatic spread is common at time of diagnosis [3–5].

Activation of programmed cell death (apoptosis) is one strategy of novel therapeutic approaches to overcome chemoresistance of tumor cells. In the extrinsic apoptosis pathway specific ligands (e.g. FASL, TNF-α or TRAIL) activate death receptors on the cell surface thereby initiating cytosolic formation of complex II containing caspase-8 resulting in their activation and caspase-3 consecutively [6]. The mitochondrial or intrinsic pathway of apoptosis is activated by intracellular signals that can be initiated by cytotoxic drugs used in chemotherapy [7]. This results in decrease of mitochondrial membrane potential (MMP) and release of mitochondrial proteins like cytochrome C, that mediates subsequent activation of caspase-9 and caspase-3, or IAP (inhibitor of apoptosis proteins) antagonist Smac (second mitochondria-derived activator of caspases).

One of the hallmarks of cancer is apoptosis deregulation that is associated with neoplastic transformation [8]. IAPs are essential regulators of apoptosis that are overexpressed in different types of cancers where they are presumed to contribute to bad outcome [9]. X-linked IAP (XIAP) inhibits intrinsic and extrinsic apoptosis initiation by direct binding to caspases-3/-7/-9 thus preventing their activation [10]. Cellular IAP (cIAP) proteins facilitate ubiquitylation of RIP1 (Receptor-interacting kinase 1) and ubiquitylation and proteasomal degradation of NIK (NF-κB-inducing kinase) and are thereby involved in regulation of canonical and non-canonical NF-κB signaling [11].

The intrinsic inhibitor Smac antagonizes the antiapoptotic effects of IAPs by interaction of its N-terminal AVPI binding motif with their BIR2 and BIR3 domains [12]. Based on this binding motif small molecule IAP antagonists (Smac mimetics) were developed to abrogate the inhibition of apoptosis induction caused by IAP overexpression commonly found in tumor cells. In several different tumor entities Smac mimetics (SM) have demonstrated synergistic sensitization for chemotherapy [13, 14]. In neuroblastoma we could show *in vitro* and *in vivo* that SM LBW242 and LCL161 were able to significantly increase the impact of cytotoxic drugs used for standard therapy [15, 16]. Interestingly, vinca alkaloids displayed the by far strongest effect if combined with SM [16]. The potential of SM for the treatment of resistant malignant diseases is currently evaluated in several clinical trials [17]. More than 20 trials are registered to date (www.clinicaltrials.gov) that investigate SM alone or combined with chemotherapy, most of them using LCL161.

## RESULTS

We demonstrated that Smac mimetic LCL161 sensitizes neuroblastoma (NB) cell lines for chemotherapy, particularly for the vinca alkaloid vincristine (VCR), *in vitro* and *in vivo* [15, 16]. LCL161 has demonstrated good tolerability in humans and mice and is currently evaluated in multiple clinical trials (www.clinicaltrials.gov) [18, 19]. In the current study we evaluated the molecular mechanisms involved in sensitization of neuroblastoma for VCR-induced apoptosis by LCL161 that so far remained obscure.

### LCL161 augments vincristine-induced caspase activation and caspase-dependent apoptosis that are accompanied by cell cycle arrest and reduced migratory potential

**Figure 1:**
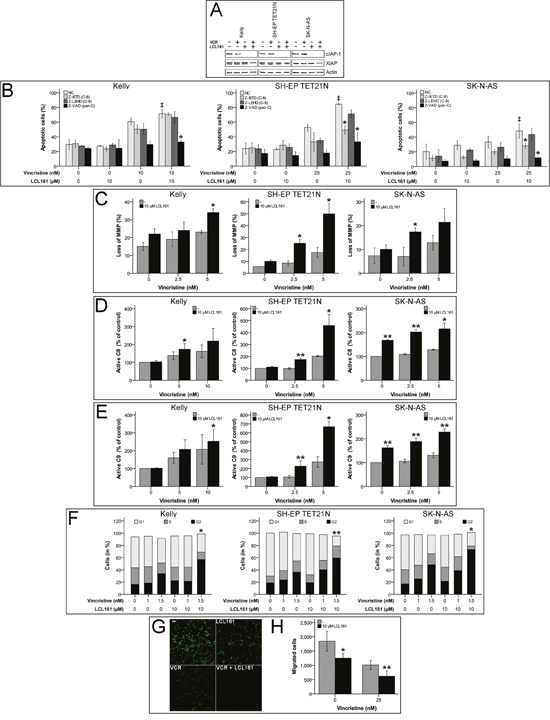
Smac mimetic LCL161 cooperates with vincristine to induce activation of extrinsic and intrinsic apoptosis and caspase-dependent cell death, G2 cell cycle arrest and reduction of migratory potential in neuroblastoma cell lines Neuroblastoma cell lines were treated with the indicated concentrations of vincristine, LCL161, and 50 μmol/L caspase inhibitors. Expression of cIAP-1, XIAP and β-actin was detected by Western Blot analysis 48 h after treatment initiation **(A)** (representative images of at least three independent Western Blots are shown). The proportions of apoptotic cells **(B)** (‡; p≤ 0.05 (NC/VCR-treated vs. NC/VCR/LCL161-treated), ^*^; p≤ 0.05 (NC/VCR/LCL161-treated vs. caspase inhibitor/VCR/LCL161-treated)), and cells with decreased mitochondrial membrane potential (MMP) **(C)** were determined by flow cytometry 48 hours after treatment initiation. Activation of caspases-8 **(D)** and -9 **(E)** was detected by Caspase-Glo assays 24 h following start of treatment. Flow cytometric cell cycle analysis was performed using propidium iodide DNA staining at 24 h time point **(F)**. Migrated cells 24 h post treatment start were visualized by fluorescence microscopy **(G)** and quantified using ImageJ and nucleus counter plugin **(H)**. Values represent the mean ± SD of three independent experiments. (C-H) ^*^p ≤ 0.05; ^**^p≤ 0.01 (VCR vs VCR/LCL161).

Induction of apoptosis by SM is proposed to be mediated by XIAP inactivation and cIAP-1 depletion, consequently we could demonstrate rapid degradation of cIAP-1 by LCL161 in neuroblastoma [14, 16, 20–22]. Treatment of neuroblastoma cell lines with VCR and LCL161 again showed degradation of cIAP-1 by LCL161, as expected XIAP protein levels were only marginally influenced (Figure 1A).

In order to characterize the molecular pathways participating in VCR/LCL161-mediated apoptosis, we analyzed involvement of intrinsic and extrinsic apoptosis. For this purpose abrogation of VCR/LCL161-induced cell death by caspase-inhibition as well as activation of caspases-8 and -9 and decrease of mitochondrial membrane potential (MMP) were determined following treatment with VCR and/or LCL161. In the cell line Kelly sensitization for vincristine-induced apoptosis by LCL161 was similarly reduced by inhibition of caspases-8 and -9, respectively. Treatment of SH-EP TET21N and SK-N-AS cells with caspase-8 inhibitor resulted in a more pronounced reduction of VCR/LCL161-induced apoptosis than that evoked by blockade of caspase-9. These findings were consistent with pan-caspase inhibition by Z-VAD, which completely abrogated the effects of LCL161 in all cell lines (Figure 1B).

Loss of MMP (ΔΨ_m_) and activation of caspase-9 are characteristics of the complex processes associated with activation of the intrinsic pathway of apoptosis, which is related to cell death induced by chemotherapy [23]. Treatment of NB cell lines with VCR induced an increase of cells with reduced MMP (Figure 1C). Combination of VCR with LCL161 significantly augmented the percentage of cells with lost MMP suggesting a VCR/LCL161-mediated activation of intrinsic apoptosis.

Activation of initiator and executioner caspases were determined in VCR/LCL161-treated cells 24 h following initiation of treatment. LCL161 significantly triggered VCR-induced cleavage of caspase-8/9 to active fragments in all cell lines showing involvement of intrinsic and extrinsic pathways of apoptosis in VCR/LCL161-mediated cell death (Figure 1D and 1E). Interestingly and in contrast to cell lines SH-EP TET21N and Kelly treatment with LCL161 induced an increase of basal caspase-8 and -9 activity >60% in SK-N-AS cells. This finding correlates with the potential of LCL161 to induce weak apoptosis induction in this cell line.

Further elucidation of the underlying mechanisms responsible for VCR-sensitization of NB by LCL161 was carried out by analysis of cell cycle profiles and migratory potential. LCL161 remarkably enhanced the VCR-induced G2 arrest (Figure 1F) and significantly decreased the amount of SK-N-AS cells that migrated following VCR treatment (Figure 1G and 1H).

### Blockade of canonical NF-κB signaling by LCL161 is dispensable for VCR/LCL161-induced apoptosis

**Figure 2:**
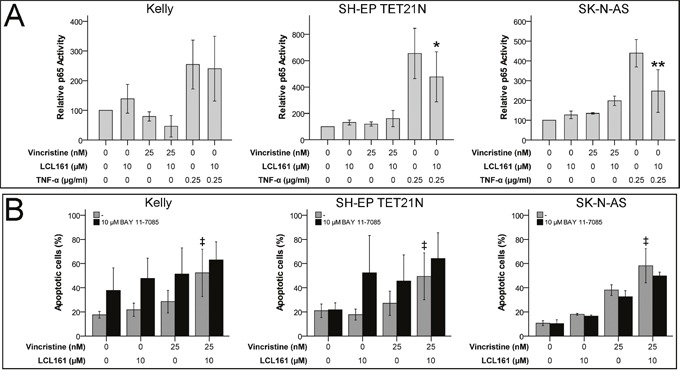
Canonical NF-κB signaling is expendable for LCL161-mediated sensitization for vincristine-induced apoptosis Neuroblastoma cell lines were treated with the indicated concentrations of vincristine, LCL161 and TNF-α. 24 h after treatment initiation p65 activity was quantified by ELISA **(A)**. Cells were additionally treated with BAY 11-7085 and apoptosis was determined by flow cytometry (Annexin V and PI staining) after 48 h **(B)**. Values represent the mean ± SD of three independent experiments. ^*^p≤ 0.05 and ^**^p≤ 0.01 (TNF-α vs. TNF-α/LCL161); ‡p≤ 0.05 (VCR vs. VCR/LCL161).

Activation of pro-survival canonical NF-κB pathway was described to be blocked during SM-induced apoptosis [17]. IκBα-mediated increase in p65 (RelA) activity is a characteristic of canonical NF-κB signaling. In neuroblastoma cell lines we detected no activation of canonical NF-κB by VCR and consequently no relevant influence on p65 activity by the combination of VCR and LCL161. In contrast, p65 activation by TNF-α was significantly reduced by LCL161 in the cell lines SH-EP TET21N and SK-N-AS (Figure 2A). Specific inhibition of canonical NF-κB activation using BAY 11-7085 did not influence the LCL-mediated sensitization of established and *de novo* neuroblastoma cell lines for VCR-induced apoptosis (Figure 2B and Supplementary Figure 1A), though. Ability of BAY 11-7085 to block canonical NF-κB was demonstrated by reduced TNF-α-mediated p65 activation following BAY 11-7085 treatment (Supplementary Figure 2A).

### LCL161-induced activation of non-canonical NF-κB cannot account for VCR/LCL161-induced apoptosis

**Figure 3:**
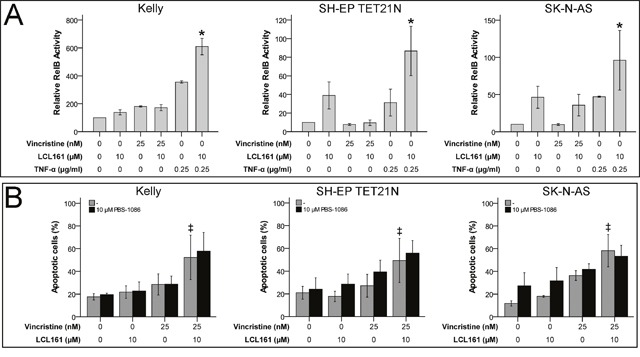
LCL161-mediated sensitization for vinca alkaloid-induced apoptosis is independent of non-canonical NF-κB signaling Neuroblastoma cell lines were treated with the indicated concentrations of vincristine, LCL161 and TNF-α. 24 h after treatment initiation RelB activity was quantified by ELISA **(A)**. Cells were additionally treated with PBS-1086 and apoptosis was determined by flow cytometry (Annexin V and PI staining) after 48 h **(B)**. Values represent the mean ± SD of three independent experiments. ^*^p≤ 0.05 (TNF-α vs. TNF-α/LCL161); ‡p≤ 0.05 (VCR vs. VCR/LCL161).

Non-canonical NF-κB activation, increase of NF-κB related TNF-α production and autocrine TNF receptor 1 (TNFR1) stimulation has been shown to be relevant for SM-related cell death [17]. We were able to display activation of non-canonical NF-κB by LCL161 as well, this was reflected by activation of RelB in cell lines SH-EP TET21N and SK-N-AS (Figure 3A). Particularly TNF-α-induced non-canonical NF-κB activation was significantly increased by LCL161 in all cell lines. Nevertheless, sensitization for VCR-induced apoptosis by LCL161 is probably unrelated to non-canonical NF-κB signaling, as LCL161 monotherapy evoked only minor effects. This presumption is supported by the finding that chemical inhibition of non-canonical NF-κB signaling using PBS-1086 (pan NF-κB inhibitor) did not impede corporate apoptosis induction of VCR and LCL161 in established and *de novo* neuroblastoma cell lines as well (Figure 3B and Supplementary Figure 1B). Inhibition of non-canonical NF-κB by PBS-1086 was validated by complete abrogation of TNF-α-induced RelB activity (Supplementary Figure 2B).

### Sensitization for VCR-induced apoptosis by LCL161 occurs independent of TNF-α

**Figure 4:**
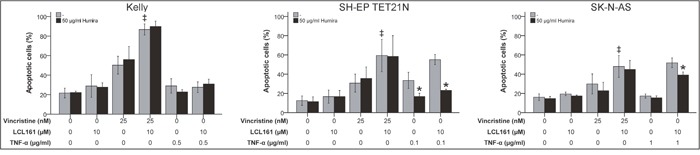
Role of TNF-α for induction of apoptosis in neuroblastoma cell lines by vincristine and its combination with LCL161 Neuroblastoma cell lines were treated with the indicated concentrations of vincristine, LCL161, TNF-α and Humira (adalimumab) and apoptosis was determined by flow cytometry (Annexin V and PI staining) after 48 h. Values represent the mean ± SD of three independent experiments. ^*^p≤ 0.05 (TNF-α vs. TNF-α/Humira and TNF-α/LCL161 vs. TNF-α/LCL161/Humira); ‡p≤ 0.05 (VCR vs. VCR/LCL161).

In the following we analyzed if TNF-α is crucial for LCL161-mediated sensitization for VCR in neuroblastoma. Thus, we detected apoptosis induction by VCR alone or in combination with LCL161 in the presence of a TNF-α-blocking antibody (adalimumab; Humira). Blocking of TNF-α had no impact on VCR-induced apoptosis as expected, but also did not impede LCL161-mediated sensitization for VCR (Figure 4 and Supplementary Figure 3). Contrary, in NB cell lines that were either sensitive for exogenous TNF-α or could be sensitized for TNF-α by LCL161 Humira significantly blocked apoptosis induction.

### RIP1-inhibition has no impact on LCL161-mediated sensitization for VCR-induced apoptosis

**Figure 5:**
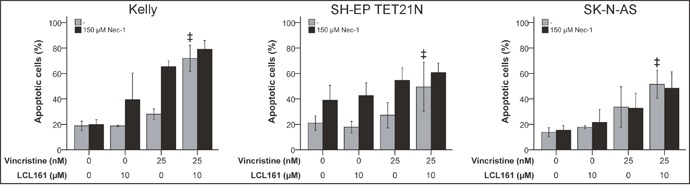
RIP1 is insignificant for LCL161-mediated sensitization for vincristine-induced apoptosis Neuroblastoma cell lines were treated with the indicated concentrations of vincristine, LCL161 and Necrostatin (Nec-1) and apoptosis was determined by flow cytometry (Annexin V and PI staining) after 48 h. Values represent the mean ± SD of three independent experiments. ‡p≤ 0.05 (VCR vs. VCR + LCL161).

Caspase-independent cell death denoted as necroptosis is dependent on the activity of the receptor interacting protein 1 (RIP1) kinase domain [24, 25]. RIP1 kinase activity is in addition essential for the assembly of the ripoptosome an alternative apoptosis complex including RIP1, cFLIP, FADD and caspase-8 that can be initiated following degradation of cIAP-1/2 by SM [26]. In order to determine the relevance of RIP1 for VCR/LCL161-induced cell death RIP1 was blocked in cells treated with VCR +/-LCL161 using chemical RIP1 inhibitor Nec-1. However, blockade of RIP1 kinase domain did not significantly affect the LCL161-mediated increase in VCR-induced apoptosis (Figure 5 and Supplementary Figure 4).

## DISCUSSION

In the current study, we analyzed the molecular mechanisms of action that account for the remarkable potency of Smac mimetic (SM) LCL161 to supplement chemotherapy of neuroblastoma [15, 16]. Here we demonstrate that SM LCL161 sensitizes for vincristine-induced apoptosis independent of NFκB, RIP1 and TNF-α.

LCL161 alone evokes sustained degradation of cIAP-1; its combination with vincristine (VCR) leads to marginal downregulation of XIAP as well. This finding has potential implications for the LCL161-mediated abrogation of apoptosis blockade that is associated with XIAP upregulation found in neuroblastoma [10, 15]. LCL161 antagonizes the binding of XIAP to caspases-3/-7/-9, an additional partial downregulation of XIAP has a probably even stronger effect that would explain the distinct apoptosis induction mediated by VCR/LCL161.

Interestingly, VCR/LCL161-induced apoptosis is effected by both activation of the intrinsic and extrinsic pathways of apoptosis and is further attended by G2 phase cell cycle arrest and reduction of migratory potential. Engagement of the mitochondrial pathway of apoptosis is reflected by a loss of the mitochondrial membrane potential (MMP) and cleavage of initiator caspase-9. Furthermore, the crucial role of the intrinsic pathway of apoptosis is demonstrated by significant reduction of VCR/LCL161-mediated apoptosis induction by caspase-9 inhibitor Z-LEHD-FMK.

NF-κB signaling has been proposed to be crucial for the observed pro-apoptotic effects of SM [17, 27, 28]. In this regard, degradation of cIAP-1/-2 is thought to be the most relevant molecular effect induced by SM leading to a suppression of the pro-survival canonical NF-κB pathway [29–31]. Thereby, TNF-α-mediated apoptosis is induced by RIP1 release from complex I to form caspase-8 activating complex II. Moreover, treatment with SM activates the non-canonical NF-κB pathway in both SM sensitive and resistant cells [17]. Remarkably, only SM sensitive cells produce TNF-α in response explaining the induced apoptosis in these cells.

Based on findings in several tumor models reaction to Smac mimetics has been categorized into three different types [32]. Smac mimetic-sensitive cells react with autocrine TNF-α synthesis and formation of complex-IIA containing RIP1 and activated caspase-8, cells of the second type are sensitized against TNF-α by Smac mimetics but do not react on Smac mimetics alone, third type cells are unresponsive against co-treatment with Smac mimetics and TNF-α [20].

In neuroblastoma we could discriminate three different tumor cell types as well, based on their TNF-α sensitivity and modulation of NF-κB pathways in response to LCL161. All cell lines had in common that they did not react with autocrine TNF-α following treatment with LCL161 (data not shown).

The first cell type (type I; Kelly) is TNF-α resistant, and besides, LCL161 is not able to induce apoptosis if combined with TNF-α. However, TNF-α-activated canonical NF-κB cannot be blocked by LCL161. TNF-α-induced non-canonical NF-κB is significantly increased by LCL161 in these type I cells, though.

The second cell type (type II; SH-EP TET21N) is sensitive for TNF-α. Here LCL161 significantly augments the apoptosis induction if combined with TNF-α. Activation of canonical NF-κB by TNF-α is significantly influenced by LCL161. Weak induction of non-canonical NF-κB is observed by LCL161 or TNF-α alone, but their combination leads to strong activation of this pathway.

In the third cell type (type III; SK-N-AS) we observed TNF-α resistance that can be overcome by LCL161. Activation of canonical NF-κB by TNF-α is efficiently blocked by LCL161. In contrast, combination of LCL161 and TNF-α leads to distinct non-canonical NF-κB activity.

Although all analyzed neuroblastoma cell lines showed differential sensitivity to Smac mimetic LCL161 and regulation of NF-κB, sensitization for VCR-induced apoptosis by LCL161 is probably independent of NF-κB and TNF-α. For this assumption several lines of evidence exist. First, activation of non-canonical NF-κB signaling (RelB activation) by LCL161 is not attended by induction of apoptosis. Second, blockade of canonical and/or non-canonical NF-κB signaling by specific repression of IκBα phosphorylation or pan-Rel inhibition had no influence on combined apoptosis induction by LCL161 and VCR. Third, treatment with TNF-α-targeting antibody adalimumab (Humira) abrogated TNF-α mediated apoptosis but did not influence LCL161-mediated and VCR-induced cell death.

Requirement of canonical and non-canonical NF**-**κB signaling for Smac mimetic-mediated apoptosis is so far not completely unraveled as several different findings indicate. General blocking of NF-κB by an IκBα super-repressor was able to prohibit sensitization of different tumors for Smac mimetics indicating that NF-κB in this context has a pro-apoptotic function [27, 28, 33]. Targeting of RelA and IKKβ by siRNA to block activation of canonical NF-κB signaling enforced Smac mimetic-induced cell death while non-canonical NF-κB was expendable [34]. Relevance of autocrine/paracrine TNF-α for Smac mimetic-induced cell death is also controversial as several findings indicate activation of both TNF-α-dependent and -independent signaling mechanisms during sensitization for chemotherapy by Smac mimetics [15, 20–22, 35–37].

RIP1 has been demonstrated to be involved in an alternative Smac mimetic-mediated mechanism leading to assembly of the ripoptosome, subsequent activation of caspase-8, and apoptosis eventually [26, 38]. This TNF-α-independent mode of action is one possible explanation for the apoptosis induction observed with Smac mimetics and chemotherapy [37, 39]. In our neuroblastoma model it turned out that RIP1 is dispensable for LCL161-mediated sensitization for VCR, as inhibition of RIP1 kinase activity using Nec-1 did not affect apoptosis induction. As RIP1 also was unnecessary for apoptosis induction of VCR and the SM BV6 it is assumable that RIP1 is in general not relevantly involved in cell death induced by the combination of SM and VCR [35].

Taken together our results again demonstrate LCL161’s high potential to supplement the cytotoxic drug VCR commonly used in chemotherapy regimen for treatment of neuroblastoma. Significant increase of VCR-induced modulation of different biological characteristics essential for cancer cell survival by LCL161 was observed. Based on the effects that were evoked by inhibition of NF**-**κB and TNF-α signaling as well as RIP-1 kinase activity it seems reasonable that augmentation of VCR-induced apoptosis depends primarily on targeting of XIAP by LCL161 and the activation of the mitochondrial pathway of apoptosis. Thus, for comprehensive characterization of the LCL161-mediated effects a more detailed analysis of the mitochondrial pathway following treatment with VCR/LCL161 is the logical next step. Furthermore, knock-down or overexpression of XIAP and/or cIAP-1 in combination with VCR would give supplementary evidence on the relevance of XIAP and cIAP expression and the IAP-directed effects of LCL161 in neuroblastoma. Additional mechanistic insights into LCL161-mediated chemosensitization will help to develop novel therapies for neuroblastoma in the future.

## MATERIALS AND METHODS

### Cell lines and cell culture

Neuroblastoma cell lines Kelly, SH-EP TET21N and SK-N-AS (purchased from American Type Culture Collection (ATCC) or German Collection of Microorganisms and Cell Cultures (DSMZ)), as well as recently established *de novo* cell lines HGW-1 and HGW-3 (kindly provided by Holger Lode; University Medicine Greifswald) were used [40]. The SH-EP TET21N system is a conditional, tetracycline-regulated MYCN expression system established in the SH-EP neuroblastoma cell line [41]. Neuroblastoma cells were maintained in RPMI 1640 or IMDM medium (HGW-1 and HGW-3) supplemented with 10-20% fetal bovine serum (both Life Technologies) and penicillin/streptomycin (10.000 U/ml / 10.000 μg/ml, Biochrom). All cells were cultivated at 37°C, 5% CO_2_-atmosphere and a relative humidity of 95%.

### Chemical compounds, biological reagents and drugs

Novartis Pharma generously provided Smac mimetic LCL161. Vincristine was obtained from Sigma-Aldrich. Inhibitors targeting caspase-8 (Z-IETD-FMK), caspase-9 (Z-LEHD-FMK) and general caspase inhibitor (Z-VAD-FMK) were purchased from R&D. Negative Control for caspase inhibitors (Z-FA-FMK) was purchased from BD Pharmingen. Combined inhibition of canonical and non-canonical NF-κB activity was performed using PBS-1086 kindly provided by Profectus BioScience. Canonical NF-κB signaling was inhibited with BAY 11-7085 obtained from Merck Millipore. Necrostatin-1 (Nec-1) from StressMarq Biosciences Inc. was used to inhibit necroptosis and RIP1 kinase activity. Working solutions were prepared by dilution of drugs with medium or ddH_2_O to designated concentrations.

### Protein extraction and Western blot analysis

Cell lysates were prepared with radioimmunoprecipitation assay (RIPA) buffer (Sigma-Aldrich) supplemented with Complete protease inhibitor cocktail and PhosSTOP – Phosphatase Inhibitor Cocktail (Roche). Standard procedures for Western blotting were followed using the following primary antibodies: cIAP-1 (AF8181; R&D), XIAP (610762; BD Biosciences) and β-actin (A1978; Sigma-Aldrich). Specific protein bands were visualized using IRDye 680RD or 800CW secondary antibodies (LI-COR) and LI-COR Odyssey infrared imaging system.

### RelB and p65 ELISA

Cells were seeded in cell culture medium in 96-well plates to adhere overnight. Cells were treated with the indicated reagents for 24 h and expression of RelB and p65 was quantified using TransAM NF-κB Family Assay Kit (43296, Active Motif) according to the manufacturer’s protocol.

### FluoroBlok migration assays

The migration assays were performed with 24-multiwell FluoroBlok cell culture inserts (Corning) with 8.0 μm pore size in 24-well plates. Cells were starved for 24 h using low-serum medium (0.2% FCS). For the assays 500 μl of cell suspension in low-serum medium was filled in the top chambers, the bottom chambers were filled with complete medium (10% FCS). Cells in the top chambers were then treated with the indicated reagents. Plates were incubated at 37°C for 24 h, medium in top chambers was removed and the 24-multiwell inserts were transferred in a 24-well plate containing 500 μl/well of 4 μg/ml Calcein AM Viability Dye (eBioscience #65-0853-39) in PBS. Inserts were incubated for 1 h at 37°C and migrated cells (fluorescently labeled) were analyzed using a fluorescence microscope at a wavelength of 495/515 nm (ex/em).

### Cell cycle analysis

Cells were seeded in cell culture medium in 24-well plates to adhere overnight. Cells were then treated with the indicated reagents for 24 h, were harvested and mixed with 200 μl of propidium iodide (PI; Invitrogen) staining solution (20 μg/ml PI in PIPES buffer (10 mM PIPES (piperazine-*N, N’*-bis(2-ethanesulfonic acid), 0.1 M NaCl, 2 mM MgCl_2_, 0.1% Triton X-100; adjusted to pH 6.8) with 200 μg/ml DNase-free RNase A). Cells were incubated at room temperature for 20 min and DNA-PI fluorescence was analyzed by flow cytometry to discriminate the G0/G1, S, G2/M phases of the cell cycle.

### Detection of apoptosis by flow cytometry

Cells were seeded in cell culture medium in 24-well plates to adhere overnight. Cells were treated with the indicated reagents for 48 h. Cells were harvested, washed twice with PBS and resuspended in Annexin V binding buffer (10 mM Hepes, 140 mM NaCl, and 0.25 mM CaCl_2_). Apoptosis was detected by Annexin V-FITC (556419; BD Pharmingen) and propidium iodide (PI) (1 mg/ml in ddH_2_O; Invitrogen) staining and flow cytometry.

### Analysis of mitochondrial membrane potential (ΔΨ_m_)

To measure the loss of the mitochondrial membrane potential (MMP), the cationic dye JC-1 (Invitrogen) was used according to the manufacturer's protocol and detected by flow cytometry.

### Detection of active caspase-8 and caspase-9 with Caspase-Glo assays

Cells were seeded in cell culture medium in 96-well plates to adhere overnight. Cells were treated with the indicated reagents for 24 h and active caspases-8 or -9 were detected using Caspase-Glo 8 and 9 assays (Promega) according to the manufacturer's protocol.

### Statistical analysis

Statistical significance of differences between experimental groups was determined using the Student t-test. A two tailed p-value ≤ 0.05 was regarded as significant.

## SUPPLEMENTARY MATERIALS FIGURES


