# Supplementary material for: Sensitization of neuroblastoma for vincristine-induced apoptosis by Smac mimetic LCL161 is attended by G2 cell cycle arrest but is independent of NFκB, RIP1 and TNF-α
Source: Oncotarget. 2017 Sep 23;8(50):87763–72. doi: 10.18632/oncotarget.21193 (PMC5675670; doi:10.18632/oncotarget.21193)
Supplement: Supplementary file 1 [file oncotarget-08-87763-s001.pdf]

Sensitization of neuroblastoma for vincristine-induced apoptosis by Smac mimetic LCL161 is attended by G2 cell cycle arrest but is independent of NFκB, RIP1 and TNF-α

SUPPLEMENTARY MATERIALS

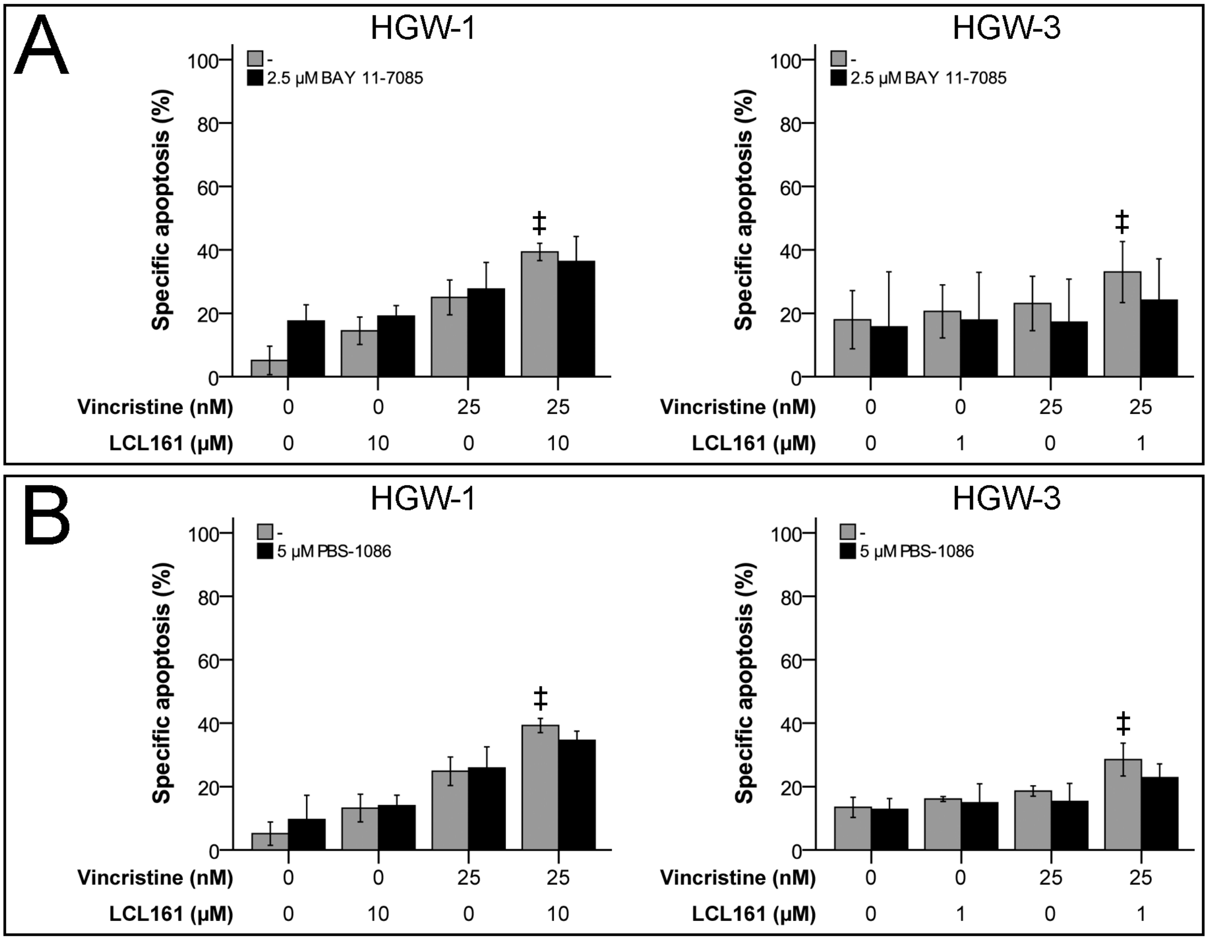

Supplementary Figure 1: NF-κB signaling cannot account for LCL161-mediated sensitization for vincristine-induced apoptosis of *de novo* NB cell lines. Neuroblastoma cells were treated with the indicated concentrations of vincristine **(A-B)**, LCL161 **(A-B)**, BAY 11-7085 **(A)** and PBS-1086 **(B)** and specific apoptosis was determined by flow cytometry (Annexin V and PI staining) after 48 h. Values represent the mean ± SD of three independent experiments. ‡p≤ 0.05 (VCR vs. VCR + LCL161).

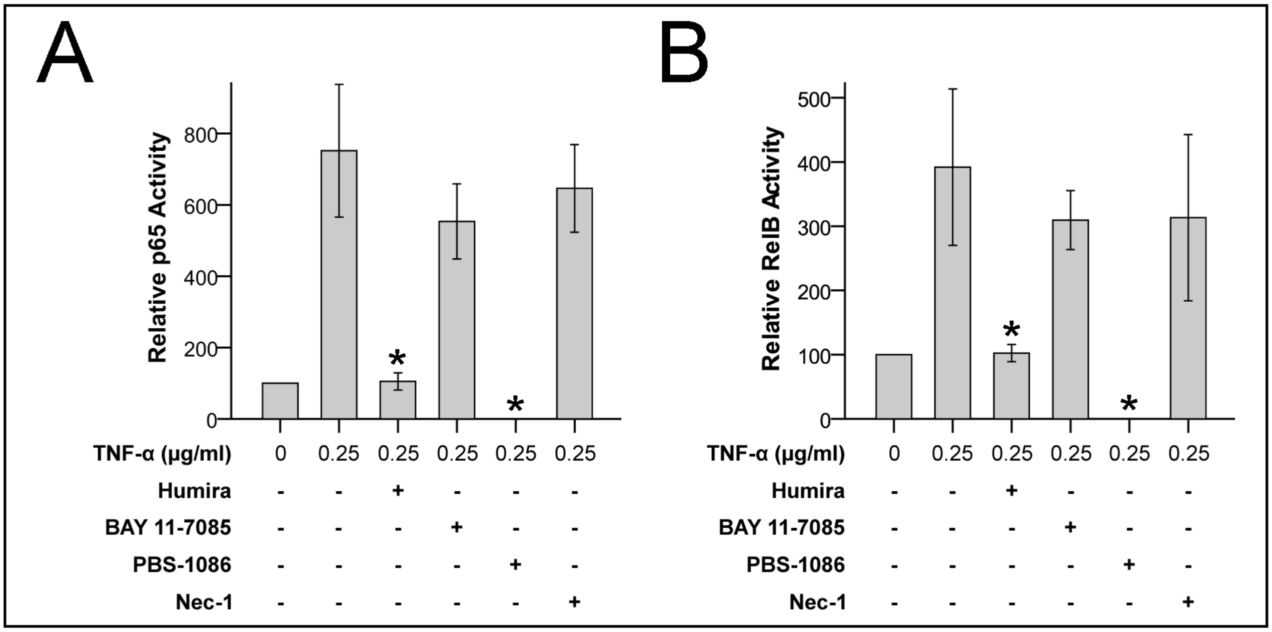

Supplementary Figure 2: Impact of inhibitors on TNF-α induced NF-κB activity. SH-EP TET21N NB cells were treated with TNF-α (0.25 µg/ml), Humira (50 µg/ml), BAY 11-7085 (10 µM), PBS-1086 (10 µM) and Nec-1 (150 µM). 24 h after treatment initiation activity of p65 **(A)** and RelB **(B)** was quantified by ELISA. Values represent the mean ± SD of two independent experiments. *p ≤ 0.05.

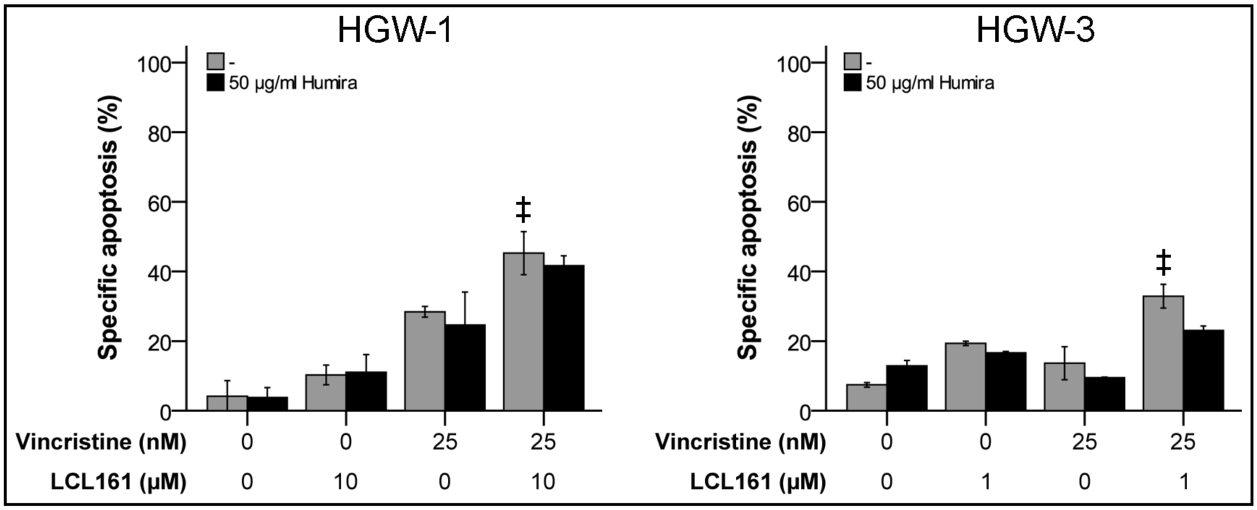

Supplementary Figure 3: Induction of apoptosis by vincristine and its combination with LCL161 in *de novo* neuroblastoma cell lines is independent of TNF-α. Neuroblastoma cells were treated with the indicated concentrations of vincristine, LCL161 and Humira and specific apoptosis was determined by flow cytometry (Annexin V and PI staining) after 48 h. Values represent the mean ± SD of three independent experiments. ‡$p \leq 0.05$ (VCR vs. VCR + LCL161).

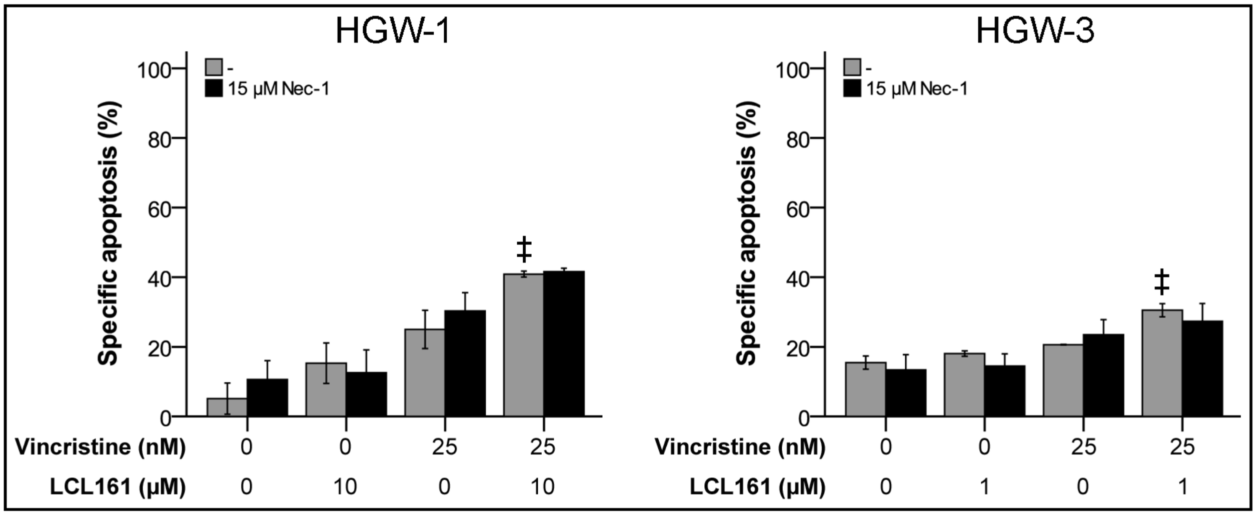

Supplementary Figure 4: LCL161-mediated sensitization for vincristine-induced apoptosis in *de novo* neuroblastoma cell lines is independent of RIP1. Neuroblastoma cells were treated with the indicated concentrations of vincristine, LCL161 and Necrostatin (Nec-1) and specific apoptosis was determined by flow cytometry (Annexin V and PI staining) after 48 h. Values represent the mean ± SD of three independent experiments. ‡$p \leq 0.05$ (VCR vs. VCR + LCL161).
